# Inactivation of the Class II PI3K-C2β Potentiates Insulin Signaling and Sensitivity

**DOI:** 10.1016/j.celrep.2015.10.052

**Published:** 2015-11-19

**Authors:** Samira Alliouachene, Benoit Bilanges, Gaëtan Chicanne, Karen E. Anderson, Wayne Pearce, Khaled Ali, Colin Valet, York Posor, Pei Ching Low, Claire Chaussade, Cheryl L. Scudamore, Rachel S. Salamon, Jonathan M. Backer, Len Stephens, Phill T. Hawkins, Bernard Payrastre, Bart Vanhaesebroeck

**Affiliations:** 1 UCL Cancer Institute, University College London, 72 Huntley Street, London WC1E 6DD, UK; 2 Inserm/UPS UMR 1048, Institut des Maladies Métaboliques et Cardiovasculaires, 1 Avenue Jean Poulhès BP 84225, 31432 Toulouse Cedex 4, France; 3 Inositide Laboratory, The Babraham Institute, Cambridge CB22 3AT, UK; 4 Mary Lyon Centre, MRC Harwell, Harwell Science and Innovation Campus, Harwell OX11 0RD, UK; 5 Department of Molecular Pharmacology, Albert Einstein College of Medicine, Bronx, NY 10461, USA

## Abstract

In contrast to the class I phosphoinositide 3-kinases (PI3Ks), the organismal roles of the kinase activity of the class II PI3Ks are less clear. Here, we report that class II PI3K-C2β kinase-dead mice are viable and healthy but display an unanticipated enhanced insulin sensitivity and glucose tolerance, as well as protection against high-fat-diet-induced liver steatosis. Despite having a broad tissue distribution, systemic PI3K-C2β inhibition selectively enhances insulin signaling only in metabolic tissues. In a primary hepatocyte model, basal PI3P lipid levels are reduced by 60% upon PI3K-C2β inhibition. This results in an expansion of the very early APPL1-positive endosomal compartment and altered insulin receptor trafficking, correlating with an amplification of insulin-induced, class I PI3K-dependent Akt signaling, without impacting MAPK activity. These data reveal PI3K-C2β as a critical regulator of endosomal trafficking, specifically in insulin signaling, and identify PI3K-C2β as a potential drug target for insulin sensitization.

## Highlights

• PI3K-C2β kinase-dead mice are viable with enhanced glucose tolerance
• PI3K-C2β inactivation sensitizes to insulin and protects from liver steatosis
• PI3K-C2β inactivation selectively enhances insulin/Akt signaling in metabolic tissues
• PI3K-C2β activity regulates very early endosomal compartments in hepatocytes

## Graphical Abstract

**Figure:**
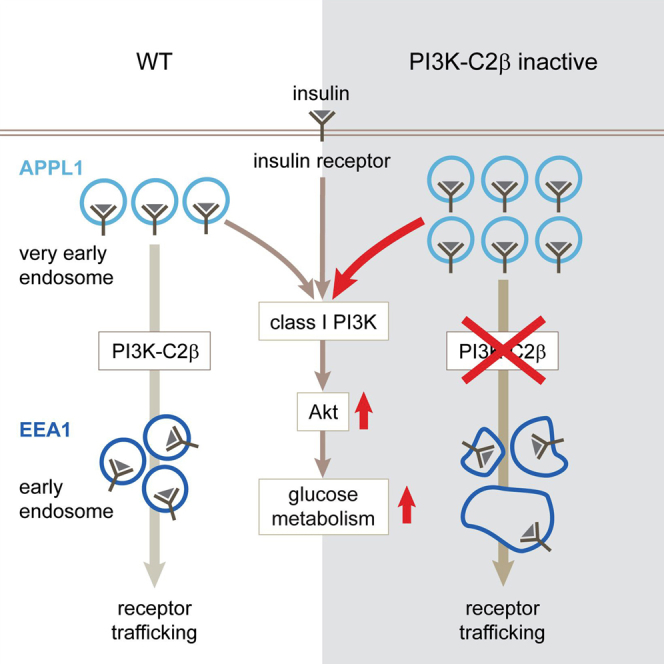

## Teaser

Organismal roles of class II PI3Ks are unclear. Alliouachene et al. show that inactivation of the class II PI3K-C2β in mice, by regulating insulin receptor trafficking, enhances insulin sensitivity and protects against high-fat-diet-induced liver steatosis. The results suggest that PI3K-C2β is a potential drug target for insulin sensitization.

## Introduction

PI3Ks, a family of lipid kinases that are activated by growth factors, hormones, and cytokines play key roles in cell growth, proliferation, and differentiation (Jean and Kiger, 2014, Vanhaesebroeck et al., 2010). Mammals have eight isoforms of PI3K, divided into three classes of which the class I PI3Ks have been most extensively studied. Through their non-redundant roles in plasma membrane receptor signaling, these PI3Ks have been implicated in overgrowth, cancer, metabolic disease, and inflammation (Jean and Kiger, 2014, Vanhaesebroeck et al., 2010). Class I PI3Ks convert the phosphatidylinositol(4,5)bisphosphate [PI(4,5)P_2_] lipid at the plasma membrane to PI(3,4,5)P_3_, also known as PIP_3_. PIP_3_ and its metabolite PI(3,4)P_2_ bind and modulate the activity of pleckstrin homology (PH) domain-containing effectors such as protein kinases (including Akt and Btk), adaptor proteins, and regulators of small GTPases. Among the four class I PI3Ks isoforms (p110α, β, γ, and δ), p110α has been identified as the most important isoform in systemic or hepatic insulin signaling (Foukas et al., 2006, Knight et al., 2006, Sopasakis et al., 2010), although within the hypothalamus both p110α and p110β are required for normal energy homeostasis (Al-Qassab et al., 2009, Tups et al., 2010).

The class II (PI3K-C2α, -C2β, and -C2γ) and III (vps34) PI3K isoforms are thought to mainly convert PI to PI3P on endosomal and autophagic membranes, resulting in the recruitment and activation of effector proteins containing FYVE or PX lipid-binding domains. Class II PI3Ks might also convert PI(4)P to PI(3,4)P_2_ (Nigorikawa et al., 2014, Posor et al., 2013). Class II PI3Ks have been reported to be activated by a wide range of agonists, such as growth factors, G protein-coupled receptors, and adhesion molecules (reviewed in Falasca and Maffucci, 2012, Jean and Kiger, 2014, Vanhaesebroeck et al., 2010). However, the molecular details of how class II PI3Ks couple to this multitude of upstream receptors remain unclear.

Previous cell-based studies have implicated a role for class II PI3Ks in the regulation of a broad variety of biological activities, including glucose transport, neurosecretory granule release, insulin secretion, endocytosis and muscle cell contraction (PI3K-C2α), cell migration and K^+^ channel activation (PI3K-C2β), and cell growth and survival (PI3K-C2α and PI3K-C2β) (Falasca and Maffucci, 2012, Jean and Kiger, 2014). The organismal roles of the class II/III PI3Ks remain less clear, with homozygous deletion of PI3K-C2α or vps34 being embryonic lethal (Franco et al., 2014, Yoshioka et al., 2012, Zhou et al., 2011) and mice homozygous deletion of PI3K-C2β being viable without reported phenotypes (Harada et al., 2005). PI3K-C2α gene knockout (KO) studies have implicated this PI3K isoform in angiogenesis (Yoshioka et al., 2012) and in the generation of the primary cilium (Franco et al., 2014). Mice homozygous for a gene-trap PI3K-C2α allele, which encodes a PI3K-C2α protein with reduced activity, are viable but develop chronic renal failure (Harris et al., 2011).

All class II/III PI3K mutant mice reported to date were created by gene targeting approaches that remove the protein of interest but do not allow the discrimination between scaffold- and kinase-dependent functions of these enzymes (Vanhaesebroeck et al., 2005). In the present study, we have therefore generated mice in which endogenous PI3K-C2β is converted to a kinase-dead protein, thereby mimicking the impact of systemically administered small molecule kinase inhibitors of PI3K-C2β. Given previous evidence from cell-based studies that class II PI3K is involved in insulin action, as shown for PI3K-C2α (Brown et al., 1999, Dominguez et al., 2011, Falasca et al., 2007, Leibiger et al., 2010, Soos et al., 2001, Ursø et al., 1999), we focused our initial characterization of the PI3K-C2β kinase-dead mice on systemic glucose homeostasis. Our data reveal a role for PI3K-C2β in the control of insulin receptor trafficking and glucose metabolism in metabolic tissues and identify this kinase as a component in the regulation of insulin signal transduction.

## Results

### Generation of PI3K-C2β Kinase-Dead Knockin Mice

**Figure 1:**
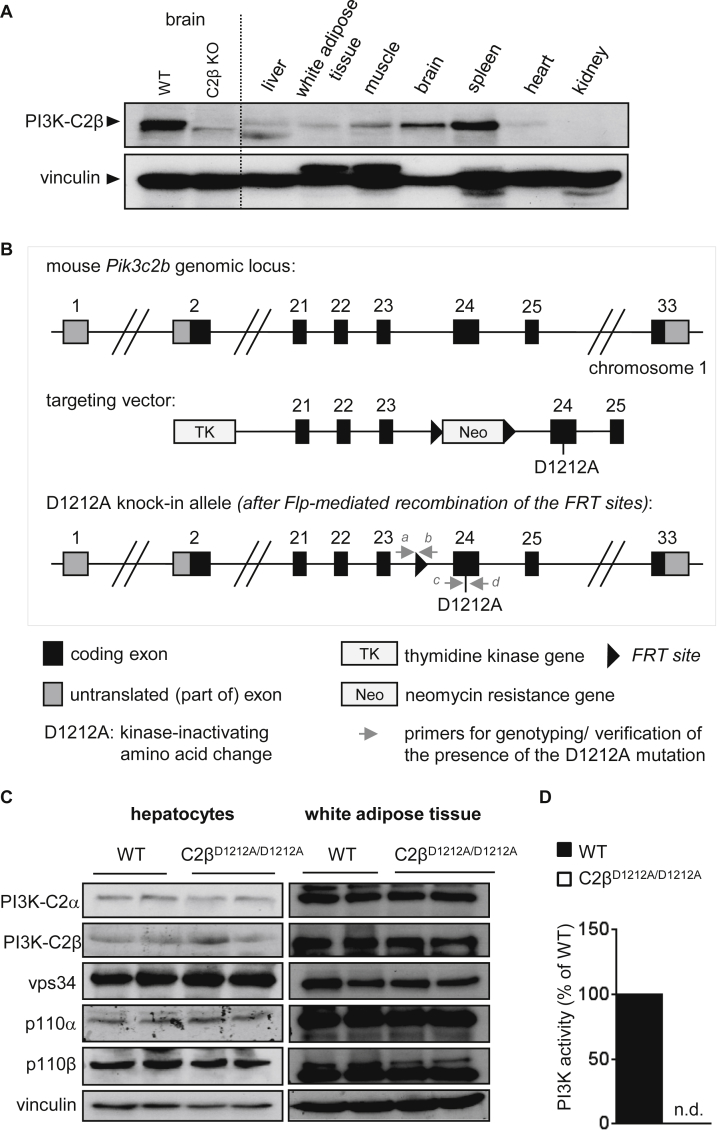
Generation and Characterization of PI3K-C2β^D1212A^ KI Mice (A) Expression of PI3K-C2β protein in mouse tissues. Brain lysates from PI3K-C2β KO mice (Harada et al., 2005) were included as controls. 100 μg of protein was loaded per lane. (B) Gene targeting strategy to generate the constitutive D1212A knockin mutation in the *Pik3c2b* gene. The D1212A mutation was introduced in the DFG motif in exon 24 of the *Pik3c2b* gene. The FRT-flanked cassette encoding *Pgk Neo* selection marker was removed in vivo by breeding onto ACTB-Flp mice. (C) PI3K isoform expression in WT and C2β^D1212A/D1212A^ mice. Homogenates of cultured hepatocytes or white adipose tissue were resolved by SDS-PAGE and immunoblotted using the indicated antibodies. Each lane represents a tissue/hepatocyte culture derived from an individual mouse. 150 and 100 μg of protein was loaded per lane for hepatocytes and white adipose tissue, respectively. (D) Lipid kinase activity associated with PI3K-C2β in WT and C2β^D1212A/D1212A^ mice. Brain homogenates were immunoprecipitated using an antibody to PI3K-C2β, followed by an in vitro lipid kinase assay using PI as a substrate. n.d., not detected.

There are currently no published data available on the tissue distribution of the PI3K-C2β protein in mice. As shown in Figure 1A, PI3K-C2β protein expression was broad and varied widely across different mouse tissues, in line with PI3K-C2β mRNA profiling studies in human tissues (Ho et al., 1997 in which PI3K-C2β is referred to as T105). Tissue distribution did therefore not provide any clear indication for a possible in vivo role of PI3K-C2β.

In order to assess the organismal role of the kinase activity of PI3K-C2β, we generated a germline knockin (KI) mouse line in which the genomic DNA encoding the aspartic acid residue on position 1212 (D1212) in the conserved ATP-binding DFG motif of PI3K-C2β was mutated to alanine (further referred to as D1212A; Figure 1B). This gene targeting strategy is expected to give rise to expression of an intact PI3K-C2β protein carrying the kinase-inactivating D1212A mutation. We previously used this strategy to constitutively inactivate class I PI3K isoforms, thereby uncovering non-redundant functions for these kinases (Vanhaesebroeck et al., 2005). Mice homozygous for the PI3K-C2β KI allele (further referred to as C2β^D1212A/D1212A^ mice) were born at a normal Mendelian ratio, with no impact on organismal growth (Figure S1) or fertility. The overall histopathology of 38 tissues from these mice was investigated and, up to 20 months of age, did not show any abnormalities (Table S1). Expression of the mutant PI3K-C2β protein and the other, non-targeted, PI3K isoforms (Figure 1C) was similar in C2β^D1212A/D1212A^ and wild-type (WT) mice, whereas the lipid kinase activity of PI3K-C2β was fully lost (Figure 1D).

### Improved Glucose Homeostasis and Insulin Sensitivity in C2β^D1212A/D1212A^ Mice

**Figure 2:**
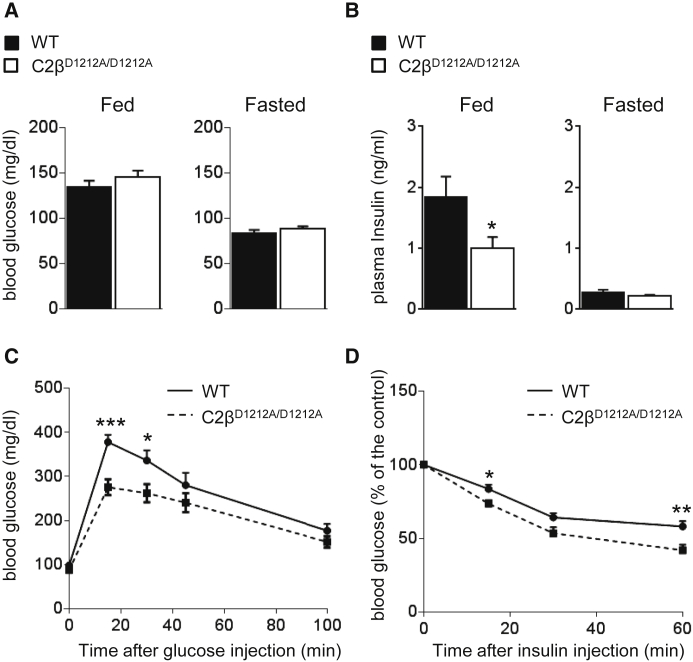
Enhanced Glucose Tolerance and Insulin Sensitivity in C2β^D1212A/D1212A^ Mice (A) Blood glucose levels under randomly fed and fasted conditions. (B) Plasma insulin levels under randomly fed and fasted conditions. (C) Glucose tolerance test after intraperitoneal injection of 2 g/kg of glucose in mice after overnight starvation. (D) Insulin tolerance test after intraperitoneal injection of 0.75 U/kg of insulin in mice after overnight starvation. Glucose levels are expressed relative to the levels in mice of the same genotype before injection of insulin. For all experiments shown, ten or more mice/genotype were used. Data represent mean ± SEM. ^∗^p < 0.05, ^∗∗^p ≤ 0.01, ^∗∗∗^p ≤ 0.001.

As part of a metabolic characterization of PI3K mutant mice, we subjected C2β^D1212A/D1212A^ mice to standard metabolic analysis. Six- to 8-week-old C2β^D1212A/D1212A^ mice had unaltered blood glucose levels under both randomly fed and fasted conditions (Figure 2A); however, the levels of circulating insulin were reduced under fed but not under fasted conditions (Figure 2B). When subjected to glucose or insulin tolerance tests, overnight fasted C2β^D1212A/D1212A^ mice showed enhanced glucose tolerance (Figure 2C), due to an increased insulin hypoglycemic response (Figure 2D). Under randomly fed conditions, WT and C2β^D1212A/D1212A^ mice had similar levels of circulating leptin, adiponectin, triglycerides, free fatty acids, and cholesterol, with similar food intake and energy expenditure (Table S2). Taken together, these data reveal that PI3K-C2β negatively regulates insulin sensitivity and glucose metabolism in vivo. Interestingly, this metabolic phenotype was observed despite the low expression of PI3K-C2β in metabolic tissues relative to other tissues, such as the spleen or brain (Figure 1A).

### PI3K-C2β Inactivation Leads to Enhanced Insulin-Stimulated Akt Signaling Selectively in Metabolic Tissues

We next investigated the impact of PI3K-C2β inactivation on insulin signaling in mice and in explanted hepatocytes. Upon insulin stimulation, the insulin receptor (IR) at the plasma membrane engages with the intracellular insulin receptor substrate (IRS) adaptor protein that recruits several cytosolic signaling proteins. These include the p85 subunit of the class I PI3Ks (leading to PIP_3_ production and activation of Akt) and the adaptor protein Grb2 that, through its association with the SOS guanine nucleotide exchange factors, activates the Ras/MAPK pathway.

**Figure 3:**
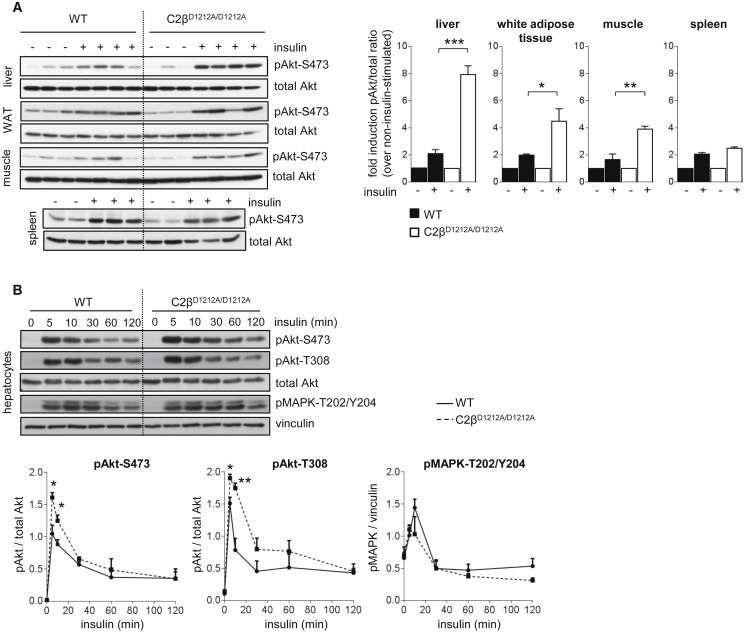
PI3K-C2β Inactivation Leads to Enhanced Insulin-Stimulated Akt Signaling Selectively in Metabolic Tissues (A) Tissue homogenates, isolated from overnight starved mice, 30 min after intraperitoneal injection of 0.75 U/kg insulin or PBS, were analyzed by SDS-PAGE and immunoblotting using the indicated antibodies. Each lane represents an individual mouse. Quantification of the signals in tissues of three to four mice/genotype is shown. WAT, white adipose tissue. (B) Cultured hepatocytes were starved overnight and stimulated for the indicated time points with 100 nM insulin, followed by SDS-PAGE analysis and immunoblotting using the indicated antibodies. Quantification of data from hepatocyte cell cultures derived from three individual mice/genotype is shown. Data represent mean ± SEM. ^∗^p < 0.05, ^∗∗^p ≤ 0.01, ^∗∗∗^p ≤ 0.001.

Compared to WT mice, stimulation of C2β^D1212A/D1212A^ mice with insulin in vivo led to enhanced Akt phosphorylation in metabolic tissues (liver, muscle, and white adipose tissue; Figure 3A). Remarkably, PI3K-C2β inactivation did not lead to an increase in insulin-induced Akt signaling in the spleen (Figure 3A), despite high expression of PI3K-C2β in this tissue compared to metabolic tissues (Figure 1A).

We next carried out a kinetic assessment of insulin signaling in cultured primary hepatocytes isolated from WT and C2β^D1212A/D1212A^ mice. Also in hepatocytes, insulin-stimulated Akt signaling was enhanced and prolonged upon PI3K-C2β inactivation (Figure 3B), with no impact on MAPK signaling (Figure 3B). Interestingly, EGF- or IGF-1-induced phosphorylation of Akt was not affected by PI3K-C2β inactivation in hepatocytes (Figures S2A and S2B).

### PI3K-C2β Inactivation Leads to Enhanced Insulin-Stimulated Class I PI3K Signaling

**Figure 4:**
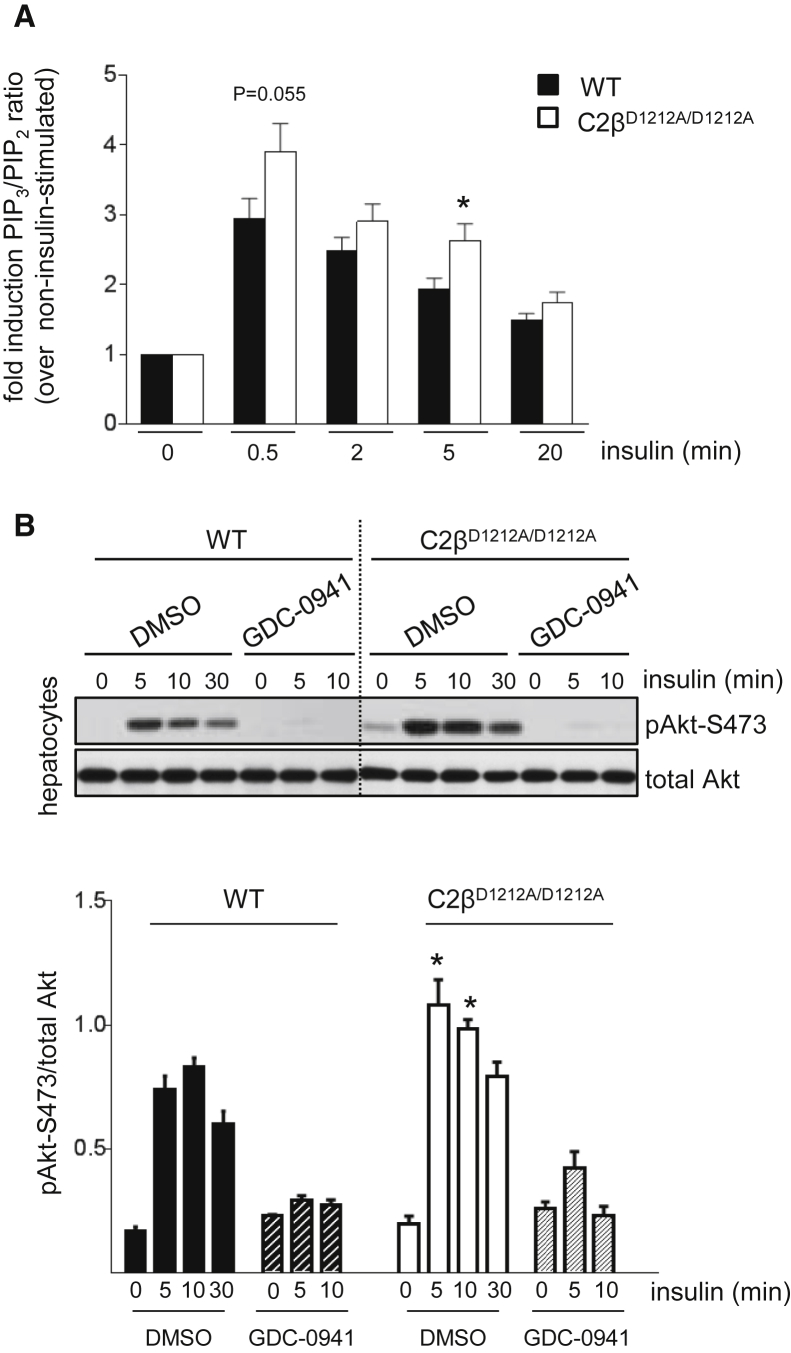
PI3K-C2β Inactivation Leads to Enhanced Insulin-Stimulated Class I PI3K Signaling (A) Hepatocytes were starved overnight and stimulated with 100 nM insulin for the indicated time points, followed by analysis of total cellular PIP_3_ levels by mass spectrometry. The mean ± SEM of four independent experiments is shown. Hepatocyte cultures from three mice/genotype were used in three of the experiments and three WT and two KI mice for the fourth experiment. (B) Hepatocytes were starved overnight and treated with 500 nM GDC-0941 for 30 min before stimulation with insulin for different time points, followed by analysis of pAkt-S473 levels. A quantification of hepatocyte cell cultures from three independent mice/genotype is shown. Data represent mean ± SEM. ^∗^p < 0.05, ^∗∗^p ≤ 0.01, ^∗∗∗^p ≤ 0.001.

Akt is activated by PIP_3_ that is produced by class I PI3Ks. In line with the enhanced activation of Akt upon PI3K-C2β inactivation (Figure 3B), insulin-stimulated PIP_3_ levels were increased to a higher extent in C2β^D1212A/D1212A^ hepatocytes as compared to WT cells (Figure 4A). Treatment of WT and C2β^D1212A/D1212A^ hepatocytes with GDC-0941, a class I PI3K-selective inhibitor, blocked insulin-stimulated Akt activation (Figure 4B). These data indicate that PI3K-C2β inactivation in hepatocytes leads to an early and transient enhancement of class I PI3K activation upon insulin stimulation, resulting in enhanced activation of Akt.

### PI3K-C2β Inactivation Does Not Affect the Early Stages of Starvation-Induced Autophagy but Induces Defects in Endosomal Trafficking and Expansion of the APPL1 Early Endosomal Compartment

**Figure 5:**
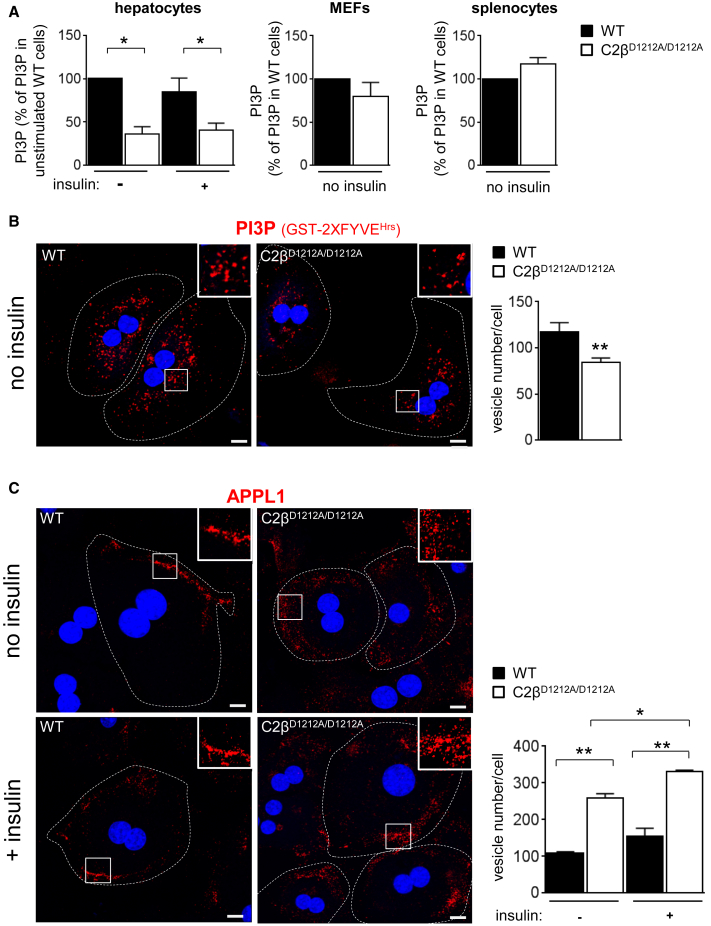
PI3K-C2β Inactivation Induces Endosomal Trafficking Defects and Expansion of the Very Early APPL1 Endosomal Compartment (A) Analysis of total cellular PI3P levels in different cell types/tissues. Hepatocytes and MEFs were starved overnight, and hepatocytes were stimulated with or without insulin (100nM) for 10 min. Splenocytes were collected from mice after overnight starvation. Cell cultures from five mice/genotype were used for all tissues, except for MEF (three WT/six C2β^D1212A/D1212A^). (B) Analysis of endogenous PI3P in fixed overnight starved hepatocytes by staining using a GST-2xFYVE^HRS^ probe. (C) Analysis of APPL1 staining in overnight starved hepatocytes stimulated with or without insulin (100nM) for 10 min. (B and C) DAPI-stained nuclei are shown in blue. The inset shows a higher magnification (300×) of FYVE- and APPL1-positive vesicles. Quantification using Metamorph software was performed on three to five independent hepatocyte cell cultures/genotype. Scale bar, 20 μm. Data represent mean ± SEM. ^∗^p < 0.05, ^∗∗^p ≤ 0.01, ^∗∗∗^p ≤ 0.001.

We next set out to uncover the underlying mechanism of this temporarily enhanced insulin-induced Akt signaling, using primary mouse hepatocytes as a cell-based model. A mass assay to quantitate PI3P levels (Chicanne et al., 2012) revealed a 60% reduction in the level of total PI3P in unstimulated hepatocytes upon inactivation of PI3K-C2β (Figure 5A). PI3P levels were unaffected by insulin stimulation in both WT and C2β^D1212A/D1212A^ hepatocytes (Figure 5A). Interestingly, PI3K-C2β inactivation did not affect the PI3P levels in mouse embryonic fibroblasts or splenocytes (Figure 5A), despite the latter expressing high levels of PI3K-C2β protein (Figure 1A).

We next assessed the subcellular distribution of PI3P in fixed hepatocytes using a GST-2xFYVE^HRS^ probe (Gillooly et al., 2000). In line with the PI3P quantitation by mass assay, unstimulated C2β^D1212A/D1212A^ cells had a clear decrease in the number of the PI3P-positive vesicles. However, there was no significant difference in the size (Figure 5B; Figure S3A) or subcellular distribution (Figure 5B) of these vesicles upon PI3K-C2β inactivation.

Key processes in which PI3P has been implicated include autophagy and endocytosis (Raiborg et al., 2013). WT and C2β^D1212A/D1212A^ hepatocytes did not show differences at the early stage of starvation-induced autophagy, as assessed by immunofluorescence (IF) staining for autophagic markers (LC3 and the PI3P-binding proteins WIPI-1 and WIPI-2; Figure S3B), indicating that PI3K-C2β does not regulate the starvation-induced autophagic PI3P pool in these cells. However, a very mild decrease in the number of WIPI-1 punctae was observed under non-starved conditions in C2β^D1212A/D1212A^ hepatocytes, compared to WT cells (Figure S3B), without any apparent impact on LC3 punctae. This suggests that PI3K-C2β may contribute to a small proportion of the PI3P pool that controls basal autophagy (i.e., the macroautophagic activity of cells in medium containing amino acids and growth factors).

PI3P has previously been reported to be important for the conversion of very early APPL1-positive endosomes into EEA1-positive endosomes (Zoncu et al., 2009). In accordance with the observed reduction in PI3P in unstimulated conditions, the number, but not the overall size, of the APPL1-positive punctae was increased in C2β^D1212A/D1212A^ hepatocytes, compared to WT cells (Figure 5C; Figure S3A). In addition, due to the “expansion” of this endosomal compartment in C2β^D1212A/D1212A^ hepatocytes, the distribution of the APPL1-positive vesicles was less restricted to the cell periphery, but instead the vesicles seemed more dispersed toward the cytoplasm (Figure 5C, inset). Under these conditions, EEA1-positive early endosomes and Rab7-positive late endosomes were found to be irregularly shaped in C2β^D1212A/D1212A^ hepatocytes, and in the case of EEA1 endosomes also enlarged, whereas their numbers were unchanged (Figure S4). Upon insulin stimulation, C2β^D1212A/D1212A^ hepatocytes resulted in a further increase in the number of APPL1-positive vesicles, with a less clear impact in WT cells (Figure 5C).

Taken together, these data suggest a defect in endosomal trafficking upon PI3K-C2β inactivation in hepatocytes, starting at the level of the very early APPL1 compartment, which could affect its maturation into EEA1-positive endosomes.

### PI3K-C2β Inactivation Increases IR Levels and Delays IR Trafficking

PI3P depletion in cultured mammalian cell lines (COS7, HeLa), and the ensuing expansion of the very early APPL1 compartment, has been shown to result in an accumulation of the EGF receptor (EGFR) in this compartment (Zoncu et al., 2009) from which endocytosed cell surface receptors can continue to signal (Platta and Stenmark, 2011). We therefore assessed the impact of PI3K-C2β inactivation on the expression and function of the IR, EGFR, and transferrin receptors in primary hepatocytes.

**Figure 6:**
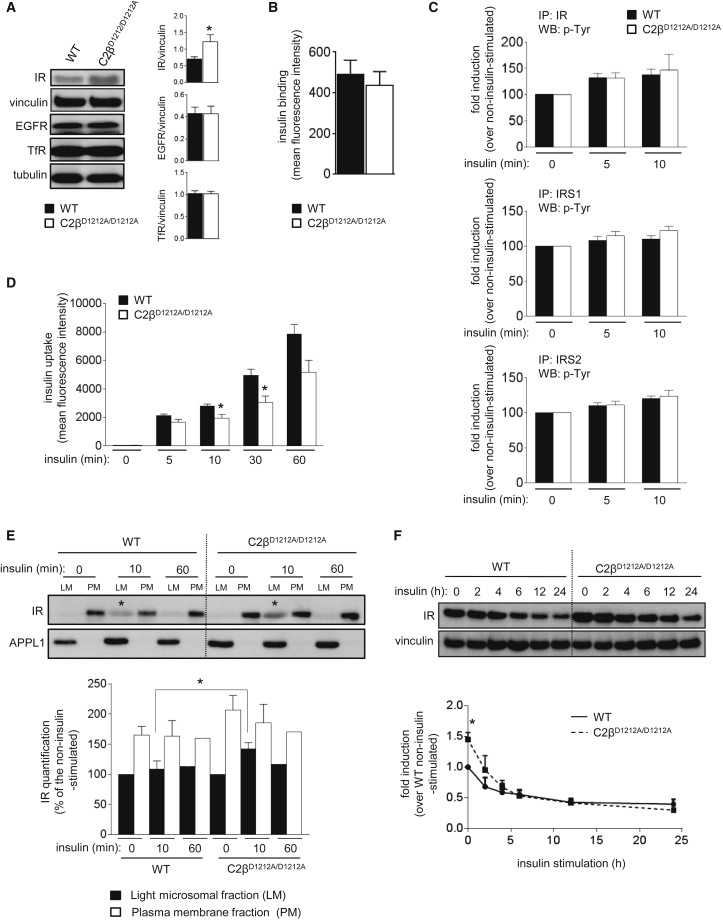
Impact of PI3K-C2β Inactivation on IR Levels and Trafficking in Hepatocytes (A) Expression levels of IR, EGF, and transferrin receptor in total cell extracts. Homogenates of hepatocytes were analyzed by SDS-PAGE and immunoblotting using indicated antibodies. Quantification of three to five independent hepatocyte cultures/genotype is shown. (B) Insulin binding capacity of hepatocytes. Quantification of three independent hepatocyte cultures/genotype is shown. (C) Cultured hepatocytes were serum-starved overnight and stimulated for the indicated times with 100 nM insulin, followed by immunoprecipitation using the indicated antibodies. The immune complexes were analyzed by western blot and probed with the indicated antibodies. The bar charts represent the quantification of western blots from four to five independent experiments, shown in Figure S6 as follows: time point 0: averages of experiments a–e (apart from IRS1, average of experiments a–d); time point 5 min: averages of experiments b–e; time point 10 min: averages of experiments a–c. (D) Insulin uptake in hepatocytes. Quantification of five independent hepatocyte cultures/genotype is shown. (E) Distribution of IR and APPL1 in subcellular fractions of hepatocytes. Hepatocytes were starved overnight and stimulated with insulin for the indicated time points, followed fractionation of lysates in light microsome (LM) and plasma membrane (PM) fractions by ultracentrifugation, followed by SDS-PAGE analysis and immunoblotting with antibodies to the indicated proteins. A representative experiment is shown. The star indicates the different levels of IR in the APPL1-positive fraction in WT and C2β^D1212A/D1212A^ cells stimulated for 10 min with insulin. Three independent hepatocyte cell cultures/genotype were used. (F) Insulin-stimulated IR degradation in hepatocytes. Cells were starved overnight, stimulated with insulin for the indicated time points, followed by SDS-PAGE analysis and immunoblotting using antibodies to IR. Three independent hepatocyte cell cultures/genotype were used. Data represent mean ± SEM. ^∗^p < 0.05, ^∗∗^p ≤ 0.01, ^∗∗∗^p ≤ 0.001.

Total cell extracts of unstimulated C2β^D1212A/D1212A^ hepatocytes showed increased expression of IR protein compared to WT (Figure 6A), with no changes in the levels of IR mRNA (Figure S5) or EGFR and transferrin receptor protein levels (Figure 6A). To assess whether this increase in total IR levels was due to higher levels of IR at the cell surface or an accumulation in intracellular compartments, we analyzed the subcellular distribution of the IR in WT and C2β^D1212A/D1212A^ hepatocytes. When incubated with labeled insulin at 4°C, WT, and C2β^D1212A/D1212A^ hepatocytes showed a similar binding capacity of insulin (Figure 6B), indicative of similar levels of IR at the cell surface. Insulin-induced tyrosine phosphorylation of IR was unaffected by PI3K-C2β inactivation (Figure 6C; Figure S6). Insulin-induced tyrosine phosphorylation of IRS1 and IRS2 was variable, and, although a tendency for an increase was observed for IRS1 in C2β^D1212A/D1212A^ mice, this was not statistically significant (Figure 6C; Figure S6). Taken together, these data suggest that the overall increase in the total levels of IR observed upon PI3K-C2β inactivation (Figure 6A) is due to an increase in the pool of *intracellular* IR that is not available for insulin binding at the plasma membrane.

Despite the unchanged levels of IR at the plasma membrane, insulin uptake at 37°C was reduced by 20% in C2β^D1212A/D1212A^ hepatocytes, especially at the 10- and 30-min time points (Figure 6D), suggesting a delay in IR trafficking early after insulin stimulation upon PI3K-C2β inactivation. This was also suggested by subcellular fractionation of C2β^D1212A/D1212A^ hepatocytes whereby the IR was found to become temporarily (i.e., 10 min after insulin stimulation) enriched in an APPL1-positive light microsomal cell fraction (Figure 6E). Upon insulin stimulation, the IR is known to mainly recycle and not to undergo acute degradation (Gorden et al., 1989, Knutson, 1991). Indeed, in WT hepatocytes, IR degradation occurred over several hours upon insulin stimulation (Figure 6F). In C2β^D1212A/D1212A^ cells, the total levels of IR normalized to WT levels in 6–7 hr (Figure 6F), suggesting a “reset” of total IR levels by ligand-induced repeated endocytic cycling, possibly as a consequence of a small fraction of the IR pool being trafficked along the degradative route in each endocytic cycle. In contrast to the IR, PI3K-C2β inactivation did not affect transferrin uptake (Figure S7).

Taken together, these data indicate that PI3K-C2β inactivation does not alter IR levels at the plasma membrane but affects IR trafficking, correlating with a temporary amplification of insulin-stimulated class I PI3K/Akt signaling.

### C2β^D1212A/D1212A^ Mice Are Protected against High-Fat-Diet-Induced Steatosis

**Figure 7:**
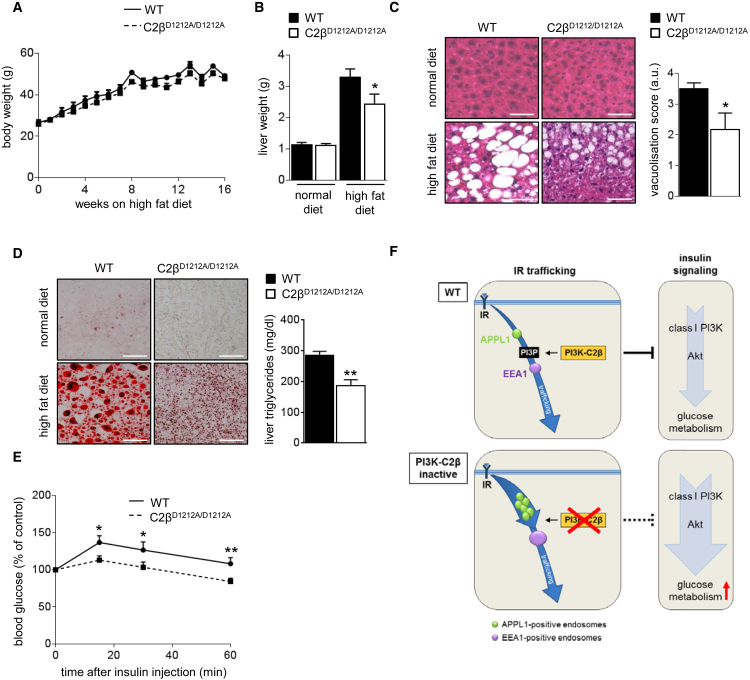
C2β^D1212A/D1212A^ Mice Are Protected against High-Fat-Diet-Induced Steatosis (A) Body weight of mice during 16 weeks of high-fat diet. (B) Liver weight after 16 weeks of high-fat diet. Five to ten livers/genotype were used of mice on a normal (control) or high-fat diet. (C) Liver histology. H&E staining of liver sections of mice after 16 weeks of high-fat diet. Quantification of vacuolization of seven livers/genotype is shown on the right. a.u., arbitrary units. Scale bar, 50 μm. (D) Oil red O staining and liver triglyceride levels. Oil red O staining of liver sections after 16 weeks of normal or high-fat diet. Ten mice/genotype. Scale bar, 50 μm. (E) Insulin tolerance test after intraperitoneal injection of 0.75 U/kg of insulin in overnight starved mice. Ten mice/genotype. (F) Model of the role of PI3K-C2β in PI3P production in hepatocytes and its impact on IR trafficking through endosomal compartments. In WT cells, PI3K-C2β generates a large fraction of the PI3P that controls the maturation of the very early APPL1-positive endosomes into early EEA1-positive endosomes. In WT mice, this attenuates IR signaling by enabling the transit of IR through endosomal compartments, either toward recycling or degradation. Inactivation of PI3K-C2β leads to a decrease in endosomal PI3P, which could delay the maturation of the very early APPL1-positive endosomes into early EEA1-positive endosomes. This leads to an accumulation of APPL1-positive endosomes and temporarily affects IR trafficking, correlating with an increased and more sustained Akt signaling in metabolic tissues. This increase in Akt signaling enhances insulin sensitivity and glucose metabolism in cells and mice with inactive PI3K-C2β. Data represent mean ± SEM. ^∗^p < 0.05, ^∗∗^p ≤ 0.01, ^∗∗∗^p ≤ 0.001.

To investigate the role of PI3K-C2β in a pathophysiological context, WT and C2β^D1212A/D1212A^ mice were subjected to a high-fat diet for 16 weeks. Whereas PI3K-C2β inactivation did not affect body-weight increase (Figure 7A), C2β^D1212A/D1212A^ mice showed a significant reduction in liver weight gain (Figure 7B), a significant protection against liver steatosis (Figure 7C) as well as reduced levels of neutral lipids (as documented by oil red O staining) and triglycerides in the liver (Figure 7D). C2β^D1212A/D1212A^ mice were also less insulin-resistant than WT mice (Figure 7E). Taken together, our data highlight an important role of PI3K-C2β in insulin signaling and glucose metabolism, especially in the liver.

## Discussion

### A New Model for Studying the In Vivo Role of the Kinase Activity of PI3K-C2β

No selective class II PI3K inhibitors are available. In order to assess the role of the kinase activity of the PI3K-C2β isoform of class II PI3Ks, we have created a mouse model in which this PI3K isoform has been rendered inactive by introduction of a germline KI mutation in the conserved DFG motif of the ATP-binding site. We previously used this strategy to uncover biological roles of the class I PI3Ks (Ali et al., 2004, Foukas et al., 2006, Graupera et al., 2008, Guillermet-Guibert et al., 2008, Okkenhaug et al., 2002). In contrast to PI3K gene deletion, such a KI strategy inactivates the PI3K in an inhibitor-like fashion, preserves the molecular balance of the expression of PI3K isoforms, minimizes compensatory effects, and, therefore, allows assessment of the role of the catalytic activity rather than possible scaffolding functions of the targeted PI3K.

### PI3K-C2β Inactivation Increases Insulin Sensitivity and Glucose Metabolism

Our study uncovered that kinase inactivation of PI3K-C2β in mice enhances insulin sensitivity and protects against high-fat-diet-induced hepatic steatosis. These findings were unexpected, for several reasons. First, previous studies in cell lines had mainly implicated PI3K-C2α, and not PI3K-C2β, in insulin signaling (Brown et al., 1999, Falasca et al., 2007, Leibiger et al., 2010, Soos et al., 2001, Ursø et al., 1999) and insulin secretion (Dominguez et al., 2011), although in most of these studies, the role of PI3K-C2β was not assessed. Second, it was remarkable to observe that inactivation of a kinase leads to *improved* insulin signaling and metabolic sensitization. Last, and still unexplained, is the apparent exclusive action of PI3K-C2β downstream of insulin, and its restriction to Akt signaling and metabolic tissues (discussed in more detail below).

Although we focused our experiments on hepatocytes, it is likely that the adipose tissue and muscle are also functionally involved in the global metabolic improvement seen in PI3K-C2β KI mice, as we also observed clearly enhanced insulin-induced Akt activation in these tissues. Based on the notion that insulin and activation of Akt are known to induce lipid storage in the liver (Leavens and Birnbaum, 2011), sustained hepatic Akt activation could be expected to lead to an increase in steatosis. This is also supported by observations in transgenic mice that express membrane-bound (and therefore constitutively active) Akt in the liver (Ono et al., 2003) or in mice with liver-specific deletion of PTEN (Horie et al., 2004), which also show enhanced steatosis. While it is challenging to link short-term signaling (minutes to hours) to long-term biological effects (4 months in the case of high-fat diet), it is important to mention that, in contrast to the *constitutive* over-activation of Akt in the mutant mice mentioned above, C2β^D1212A/D1212A^ mice only display a *transient* over-activation of Akt upon insulin stimulation. Moreover, it is very likely that the systemic enhancement in insulin sensitivity, with increased Akt activity in muscle and adipose tissue in addition to the liver, leads to an overall improved metabolism of C2β^D1212A/D1212A^ mice, which reduces the development of hepatic steatosis as a consequence.

Together with the observation that no abnormalities were observed in an in-depth histopathological analysis of adult C2β^D1212A/D1212A^ mice, our data identify PI3K-C2β as a potential drug target for insulin sensitization in the treatment of insulin resistance in type 2 diabetes or non-alcoholic fatty liver disease.

### PI3K-C2β Is a Major Endosomal Producer of Basal PI3P in Hepatocytes

One previous study has implicated PI3K-C2β in PI3P production, namely, in the PI3P synthesis induced by lysophosphatidic acid in HeLa and SKOV-3 cell lines (Maffucci et al., 2005). Our study shows that PI3K-C2β is required for a large fraction (60%) of the basal PI3P in hepatocytes and therefore demonstrates that class II PI3Ks can significantly contribute to PI3P pools in vivo in the liver. Interestingly, the reduction in PI3P upon PI3K-C2β inactivation selectively impacted on endosomal trafficking but did not affect the formation of starvation-induced early autophagic vacuoles in these cells, indicating that PI3K-C2β mainly controls the endosomal PI3P pool in hepatocytes. It is possible that vps34 or class II PI3K isoforms other than PI3K-C2β (Devereaux et al., 2013) generate the autophagic PI3P pool in the liver.

### PI3K-C2β Inactivation Results in an “APPL1 Expansion Signature”

IF analysis of intracellular vesicles revealed that PI3K-C2β inactivation led to an expansion of the very early APPL1-positive endosomal compartment in hepatocytes, under basal and insulin-stimulated conditions. We show in this study that PI3K-C2β is linked to the regulation of intracellular vesicular trafficking.

APPL1 is a scaffolding protein with multiple functional domains, including a Bin1/amphiphysin/rvs167 (BAR) domain, a PH domain, a phosphotyrosine binding (PTB) domain, and a CC motif (Deepa and Dong, 2009). APPL1 interacts with various receptors (such as the IR, TrkA, and the adiponectin receptor), signaling and scaffolding proteins (including Akt, IRS, and the OCRL inositol polyphosphate 5-phosphatase), and vesicular trafficking proteins (such as GTP-bound Rab5) (Deepa and Dong, 2009, Ryu et al., 2014). Our findings are in line with a cell-based study that highlighted a critical role for PI3P in the maturation of the APPL1-positive very early endosomes to EEA1-positive early endosomes (Zoncu et al., 2009). These authors showed that PI3P depletion in the Rab5-positive compartment (by introduction of PI3P-specific phosphatases) led to an accumulation of the EGFR in an expanded very early APPL1-positive endosomal compartment upon EGF stimulation, resulting in enhanced downstream signaling (Zoncu et al., 2009). Our study uncovers a similar APPL1-based mechanism for IR trafficking, correlating with enhanced insulin-stimulated Akt activation. Further support for a role for APPL1 in insulin action comes from observations made by APPL1 KO/overexpression in mice. For example, APPL1 KO mice show the opposite metabolic phenotype to C2β^D1212A/D1212A^ mice, being insulin-resistant and glucose-intolerant with decreased insulin-stimulated Akt signaling in the liver, muscle, and adipose tissue (Ryu et al., 2014). Conversely, APPL1 overexpression in the liver increases Akt activation and alleviates insulin resistance in obese mice (Cheng et al., 2009).

### PI3K-C2β Inactivation Enhances Insulin Signaling in Metabolic Tissues

Under basal conditions, C2β^D1212A/D1212A^ hepatocytes had substantially lower levels of total cellular PI3P than WT cells. Insulin stimulation of these cells did not alter PI3P levels in either genotype but did increase PIP_3_ levels, as expected. Interestingly, insulin stimulation led to a higher PIP_3_ increase in C2β^D1212A/D1212A^ hepatocytes compared to WT cells.

These data are consistent with a model (Figure 7F) whereby, in hepatocytes, PI3K-C2β is responsible for the *constitutive* production of a large fraction of the PI3P required for the basal endosomal flux. Under basal conditions, C2β^D1212A/D1212A^ hepatocytes had an ∼2-fold increase in overall IR levels over WT cells, without higher IR expression at the cell surface, pointing to the existence of an intracellular pool of IR. At present, the exact subcellular location of this IR pool is unknown. Given that the APPL1-positive compartment is expanded, it is possible that the IR is stuck as a consequence of a “traffic jam” in its endocytic flux.

Upon insulin stimulation, there was no difference in tyrosine phosphorylation of the IR, in line with unaltered IR levels at the cell surface. However, as insulin-mediated Akt signaling was temporarily enhanced upon PI3K-C2β inactivation, it is possible that the ligand-bound IR signals longer as a result of its slower endocytosis due to the “traffic jam” in the APPL1 compartment. A similar phenomenon was previously shown for the EGFR and its downstream signaling upon expansion of the APPL1 compartment due to PI3P reduction (Zoncu et al., 2009). Upon prolonged (6–7 hr) exposure to insulin, the higher overall IR levels in C2β^D1212A/D1212A^ hepatocytes were found to return to that seen in WT cells, possibly due to insulin-induced repeated endocytic cycling whereby a small fraction of the activated IR pool is trafficked along the degradative route in each endocytic cycle.

Supportive of our model that IR traffic is delayed in the APPL1 compartment is the recent report showing that APPL1 can bind both IR and IRS and facilitate the insulin-stimulated interaction between IR and IRS (Ryu et al., 2014). The capacity of IR/IRS/Akt/APPL1 to dynamically interact, in a subcellular compartment controlled by PI3P, is likely to be key in explaining the selective metabolic impact of PI3K-C2β inactivation.

Unfortunately, we were not able to visualize endogenous IR, PIP_3_, or phospho-Akt by IF using an extensive range of home-made or commercially available antibodies. IF analyses using fluorescently labeled insulin on primary hepatocytes, both on fixed cells or by live imaging, were also not successful. We therefore do not know the exact subcellular location of increased PIP_3_ production and Akt activation upon PI3K-C2β inactivation.

### Selective Role of PI3K-C2β in Insulin-Stimulated Akt Activation in Metabolic Tissues

One of the most surprising findings of our work is the apparent exclusive action of PI3K-C2β downstream of insulin, and its restriction to Akt signaling and metabolic tissues. Our data clearly show that a reduction in PI3P, as a consequence of PI3K-C2β inactivation, and the ensuing block in endosomal traffic enhances cell signaling in a very specific manner. At the organismal level, PI3K-C2β inactivation induced an increase in insulin-stimulated Akt signaling specifically in insulin target tissues (liver, muscle, and adipose tissue) but not in the spleen where PI3K-C2β is expressed highly. PI3K-C2β inactivation did also not affect the basal levels of PI3P in splenocytes or MEFs. Further studies conducted on hepatocytes showed additional specificity of PI3K-C2β action. First, at the receptor level, PI3K-C2β inactivation led to increased IR protein expression in these cells, without altering the levels of the receptors for EGF or transferrin and modulated the intracellular trafficking of the IR but not that of the transferrin receptor. Second, at the ligand level, PI3K-C2β inactivation increased insulin-induced Akt signaling in hepatocytes, without altering EGF- or IGF-mediated Akt signaling. Last, but not least, at the downstream signaling level, PI3K-C2β inactivation in hepatocytes led to an increased insulin-stimulated Akt activation without affecting MAPK signaling.

At present, we do not have a clear explanation for these observations. The absolute expression levels of the PI3K-C2β protein in different tissues are unlikely to be key in this phenomenon, given that PI3K-C2β expression is high in the spleen compared to liver. PI3P turnover in different tissues may depend on tissue-specific activities of the PI3Ks and other PI kinases but also of lipid phosphatases. It is also possible that such specificity could be provided by the scaffolding properties of APPL1, allowing this protein to interact with tissue- and receptor-specific binding partners, creating tissue-/ligand-/signaling-selective protein hubs. It is of interest to note that, despite the broad tissue distribution of APPL1, numerous studies have described APPL1 as an important player specifically in metabolic tissues and in particular in insulin signaling (Cheng et al., 2014). In line with our observations, other studies have documented that alterations in APPL1 expression can differentially affect signaling depending on the stimulus. For example, APPL1 KO in MEFs was found to reduce Akt activation induced by Hepatocyte Growth Factor, but not by EGF, insulin, or serum (Tan et al., 2010). Likewise, APPL1 knockdown in zebrafish led to a decrease in growth factor-induced Akt signaling with no impact on MAPK (Schenck et al., 2008). These data suggest that APPL1 endosomes can serve as signaling platforms for selective recruitment and activation of signaling components.

### Conclusions

Taken together, our study reports on the creation of the class II PI3K knockin mice to model systemic PI3K-C2β kinase inactivation and identifies this isoform of PI3K as a drug target for the treatment of insulin resistance in type 2 diabetes or non-alcoholic fatty liver diseases.

## Experimental Procedures

### Mice

All experiments were performed on 6- to 12-week-old male C57BL/6J mice, unless otherwise specified. Mice were kept on standard chow diet (20% protein, 75% carbohydrate, 5% fat) on a 12-hr light-dark cycle (lights on at 7 a.m.) with free access to water in individually ventilated cages and cared for according to United Kingdom Animals (Scientific Procedures) Act (1986). For high-fat-diet experiments, mice were maintained on diet 824053 from Special Diet Services (20% protein, 35% carbohydrate, and 45% fat) for 16 weeks.

### Creation of C2β^D1212A^ Mice and PCR Genotyping

Mouse gene targeting was performed by Artemis in C57BL/6NT embryonic stem cells. Mice were backcrossed on the C57BL/6J strain (Charles River Laboratories) for greater than ten generations, and mice used for experiments were on mixed C57BL/6J × C57BL/6NT background, with WT littermates used as controls. The sequences of the primers used for genotyping are as follows: forward primer (*a* in Figure 1B): 1614-29 KI: CACTGCAGGAAGTGTGAAGC; antisense primer (*b* in Figure 1B): 1614-30 KI: GTGGACAGAAAGGCTGATGC, with expected fragments of 235 (WT) and 398 (KI) bp. PCR conditions were as follows: 95°C for 5 min, 34 cycles of (95°C 30 s, 65°C 30 s, 72°C 1 min) and 72°C for 10 min. The presence of the D1212A mutation was verified by sequencing of a PCR fragment generated using forward primer 1743-25 ivm: GCTTTGGTATATGATGAAGG (*c* in Figure 1B) and antisense primer 1743-26 ivm: GTCTTCTGGTCTCCAGAAGC (*d* in Figure 1B) using the PCR conditions described above. PCRs were performed using Titanium Taq polymerase (Clontech) on a ThermalCycler (MJ Research).

### Hepatocyte and Mouse Embryonic Fibroblast Isolation and Culture

Primary mouse hepatocytes were isolated from 8- to 12-week-old mice as described, with minor changes (Guidotti et al., 2003). Briefly, primary hepatocytes were isolated by a two-step perfusion protocol using collagenase I (Sigma) and seeded on collagen-coated plates in complete medium (William’s E GlutaMAX medium containing 0.1% BSA, 1% penicillin/streptomycin, 25 nM dexamethasone [Sigma], and 100 nM insulin) in the presence of 10% (v/v) fetal bovine serum (FBS). After 4 hr incubation at 37°C to allow cell adhesion, the medium was replaced either by starvation medium (complete medium without insulin) or complete medium (for autophagy studies), and cells were further incubated at 37°C overnight.

For signaling studies, fluorescence-activated cell sorting (FACS) analysis, immunofluorescence, lipid analysis, and cell fractionation, culture medium was removed and replaced by fresh starvation medium containing insulin (100 nM), EGF (200 nM), or IGF-1 (3.9 nM) for the indicated times. In some experiments, GDC-0941 (500 nM) was added at 30 min before cell stimulation.

For autophagy studies, complete medium was removed and cultures were washed twice with amino-acid- and serum-free medium (EBSS; Invitrogen), and cells were maintained in EBSS for 30 min. Control, non-starved cells were washed in complete medium instead of EBBS.

Mouse embryonic fibroblasts (MEFs) were isolated from intercrosses of mice heterozygous for the PI3K-C2β^D1212A^ allele as described (Foukas et al., 2006).

### Antibodies and Reagents

All antibodies were against mouse proteins as follows: APPL1, EEA1, Rab7, GST, pAkt-S473, pAkt-T308 Akt, p42/44 pMAPK-T202/pY204, Akt, p110α, insulin receptor, vps34 (Cell Signaling Technology); PI3K-C2α, PI3K-C2β (BD Biosciences); p110β, ptyr99 (Santa Cruz Biotechnology); vinculin, α-tubulin, EGFR (Sigma); transferrin receptor (Abcam); and LC3 (2G6; Nanotools). Antibodies to WIPI-1 and WIPI-2 were kindly provided by Sharon Tooze (London Research Institute). In-house-made antibodies to IRS1 and IRS2 were provided by Dominic Withers (Imperial College London). Fluorescein isothiocyanate (FITC)-insulin (bovine) was from Sigma, and FITC-transferrin (human) was from Molecular Probes. Unless otherwise mentioned, PBS (Sigma) was Ca^2+^ and Mg^2+^ free. All culture media for primary cell culture were from Invitrogen. A plasmid expressing GST-2xFYVE^HRS^ (Gillooly et al., 2000) was kindly provided by Harald Stenmark, Norway. Recombinant GST protein was purified from *E. coli* BL21 (DE3) cells according to the manufacturer’s instructions. All buffers used during purification of the GST-fusion steps were EDTA free, and the recombinant protein was dialyzed against HEPES buffer (pH 7.4) containing 10 μM ZnCl_2_. Agonists used were insulin (human [Actrapid] and bovine [Sigma] for in vivo and in vitro experiments, respectively), EGF (human; PeproTech), or IGF-1 (human; PeproTech). GDC-0941 was from Axon-Medchem.

### Statistical Analysis

All data are shown as mean ± SEM. Data sets were compared for statistical significance using a two-tailed Student’s t test. All statistical analyses were generated using Excel software and statistical significance indicated as ^∗^p < 0.05, ^∗∗^p ≤ 0.01, ^∗∗∗^p ≤ 0.001.

## Author Contributions

S.A., B.B., G.C., K.E.A., W.P., K.A., C.V., P.C.L., Y.P., C.C., and R.S.S. performed experiments and data analyses with input from B.P., J.M.B., L.S., P.T.H., and B.V.; C.L.S. analyzed and interpreted histopathology. S.A., B.B., and B.V. wrote the paper.

## Supplemental Information

Document S1. Supplemental Experimental Procedures, Figures S1–S7, and Tables S1 and S2

Document S2. Article plus Supplemental Information
