# Supplementary material for: Inactivation of the Class II PI3K-C2β Potentiates Insulin Signaling and Sensitivity
Source: Cell Rep. 2015 Nov 19;13(9):1881–94. doi: 10.1016/j.celrep.2015.10.052 (PMC4675724; doi:10.1016/j.celrep.2015.10.052)
Supplement: Document S1. Supplemental Experimental Procedures, Figures S1–S7, and Tables S1 and S2 [file mmc1.pdf]

Cell Reports

Supplemental Information

Inactivation of the Class II PI3K-C2β Potentiates Insulin Signaling and Sensitivity

Samira Alliouachene, Benoit Bilanges, Gaëtan Chicanne, Karen E. Anderson, Wayne Pearce, Khaled Ali, Colin Valet, York Posor, Pei Ching Low, Claire Chaussade, Cheryl L. Scudamore, Rachel S. Salamon, Jonathan M. Backer, Len Stephens, Phill T. Hawkins, Bernard Payrastre, and Bart Vanhaesebroeck,

Figure S1

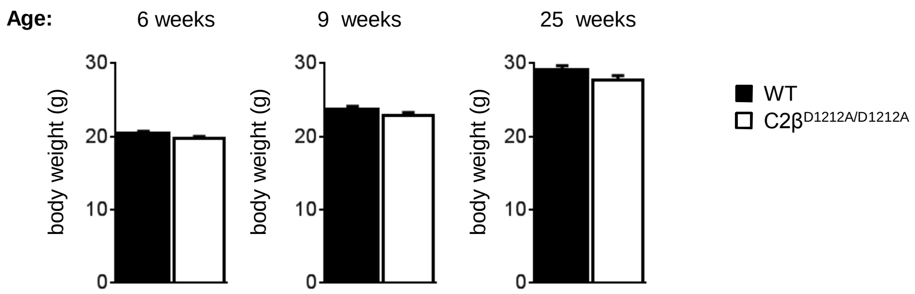
Age:
6 weeks
9 weeks
25 weeks
body weight (g)
30
20
10
0
WT
C2β D1212A/D1212A

Figure S2

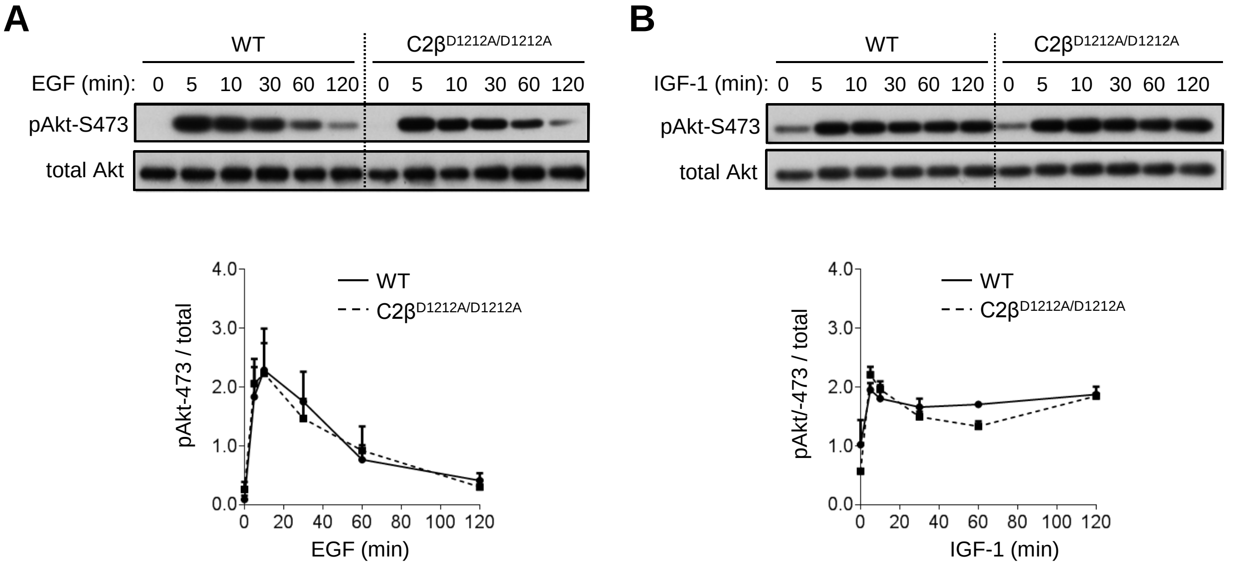
A
WT
C2β D1212A/D1212A
EGF (min): 0 5 10 30 60 120 0 5 10 30 60 120
pAkt-S473
total Akt
WT
C2β D1212A/D1212A
pAkt-473 / total
EGF (min)
4.0
3.0
2.0
1.0
0.0
0 20 40 60 80 100 120
B
WT
C2β D1212A/D1212A
IGF-1 (min): 0 5 10 30 60 120 0 5 10 30 60 120
pAkt-S473
total Akt
WT
C2β D1212A/D1212A
pAkt/-473 / total
IGF-1 (min)
4.0
3.0
2.0
1.0
0.0
0 20 40 60 80 100 120

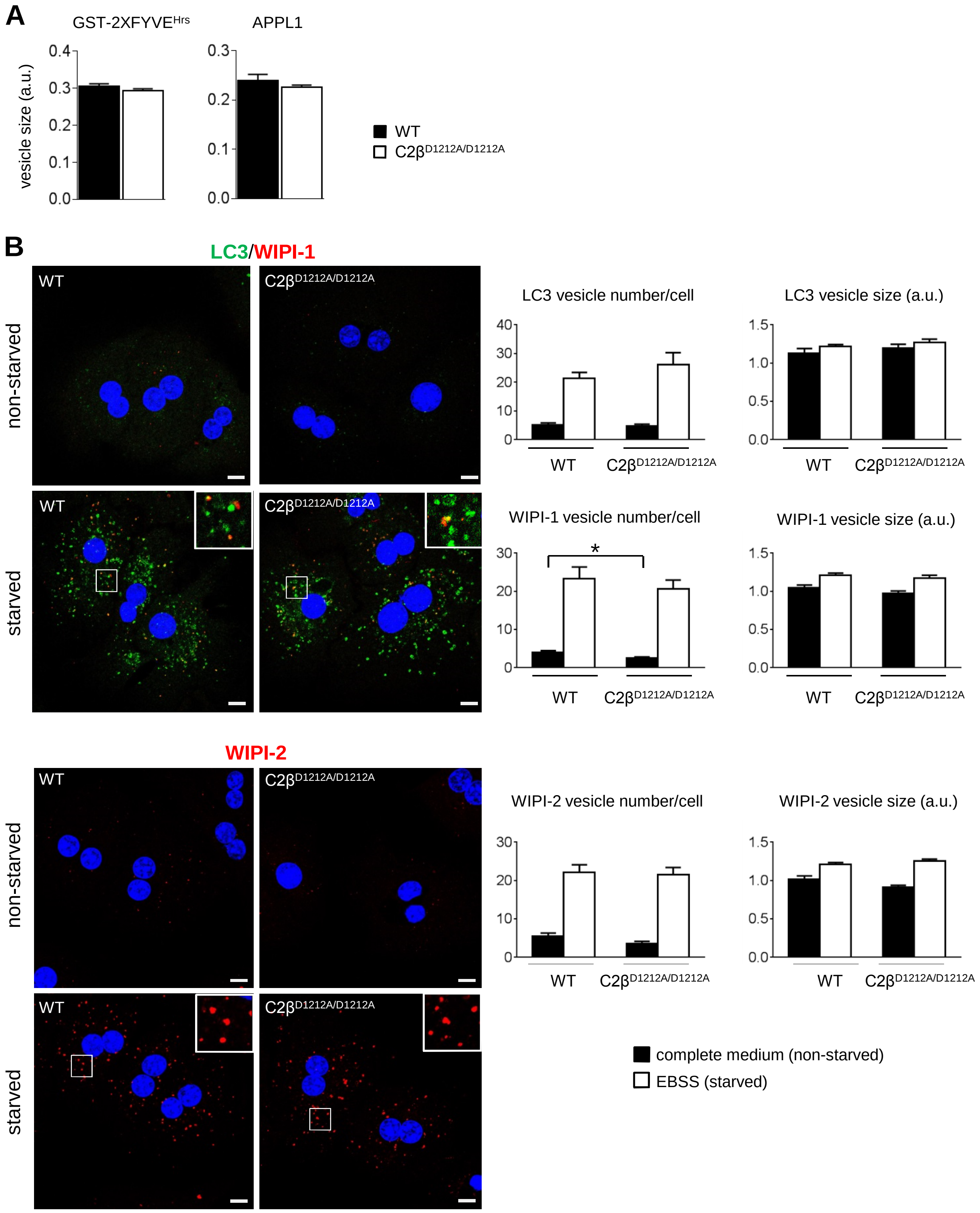
A
GST-2XFYVE^Hrs
APPL1
vesicle size (a.u.)
WT
C2β^D1212A/D1212A
B
LC3/WIPI-1
non-starved
starved
LC3 vesicle number/cell
LC3 vesicle size (a.u.)
WIPI-1 vesicle number/cell
WIPI-1 vesicle size (a.u.)
WIPI-2
WIPI-2 vesicle number/cell
WIPI-2 vesicle size (a.u.)
complete medium (non-starved)
EBSS (starved)

Figure S4

Alliouachene *et al.*

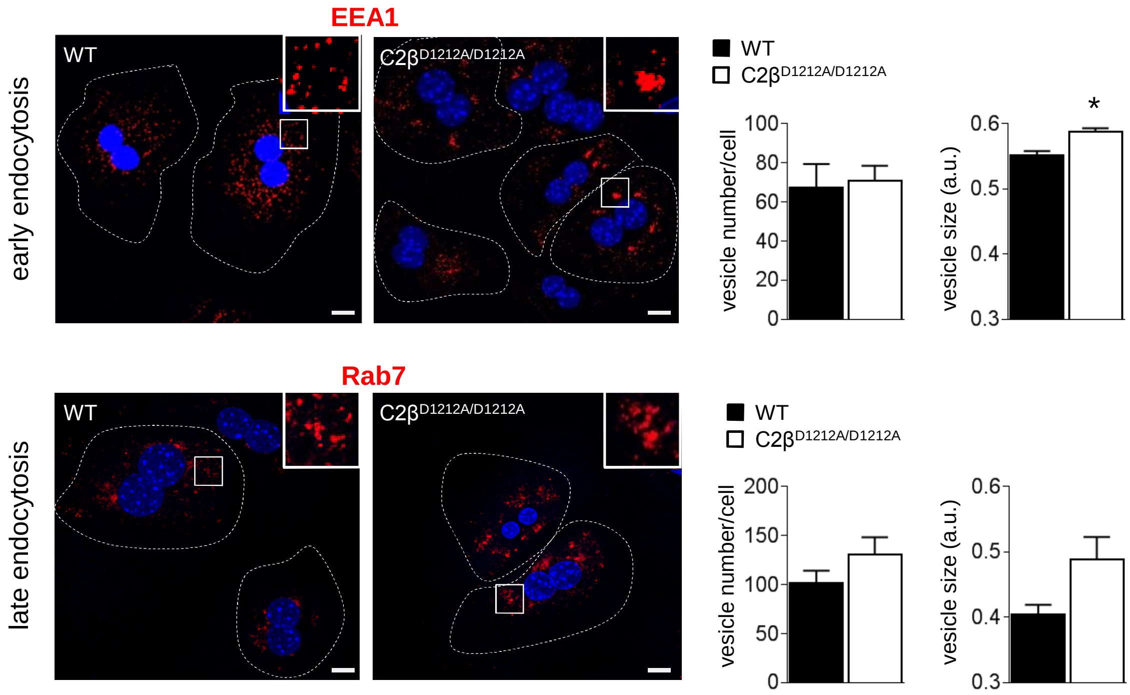

Figure S5

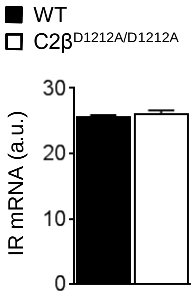

Figure S6

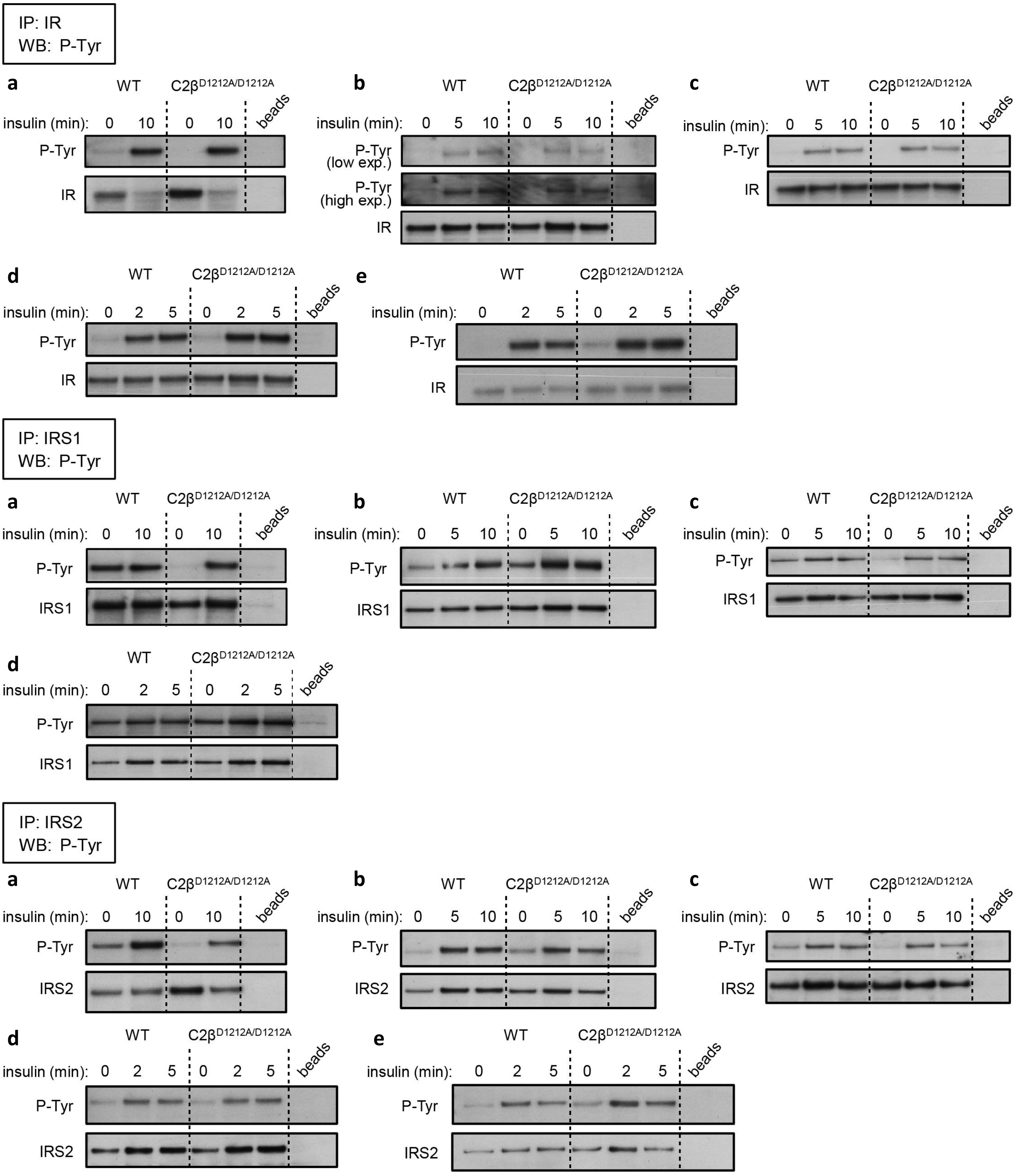

IP: IR
WB: P-Tyr
a
WT
C2β D1212A/D1212A
insulin (min): 0 10 0 10
beads
P-Tyr
IR
b
WT
C2β D1212A/D1212A
insulin (min): 0 5 10 0 5 10
beads
P-Tyr (low exp.)
P-Tyr (high exp.)
IR
c
WT
C2β D1212A/D1212A
insulin (min): 0 5 10 0 5 10
beads
P-Tyr
IR
d
WT
C2β D1212A/D1212A
insulin (min): 0 2 5 0 2 5
beads
P-Tyr
IR
e
WT
C2β D1212A/D1212A
insulin (min): 0 2 5 0 2 5
beads
P-Tyr
IR
IP: IRS1
WB: P-Tyr
a
WT
C2β D1212A/D1212A
insulin (min): 0 10 0 10
beads
P-Tyr
IRS1
b
WT
C2β D1212A/D1212A
insulin (min): 0 5 10 0 5 10
beads
P-Tyr
IRS1
c
WT
C2β D1212A/D1212A
insulin (min): 0 5 10 0 5 10
beads
P-Tyr
IRS1
d
WT
C2β D1212A/D1212A
insulin (min): 0 2 5 0 2 5
beads
P-Tyr
IRS1
IP: IRS2
WB: P-Tyr
a
WT
C2β D1212A/D1212A
insulin (min): 0 10 0 10
beads
P-Tyr
IRS2
b
WT
C2β D1212A/D1212A
insulin (min): 0 5 10 0 5 10
beads
P-Tyr
IRS2
c
WT
C2β D1212A/D1212A
insulin (min): 0 5 10 0 5 10
beads
P-Tyr
IRS2
d
WT
C2β D1212A/D1212A
insulin (min): 0 2 5 0 2 5
beads
P-Tyr
IRS2
e
WT
C2β D1212A/D1212A
insulin (min): 0 2 5 0 2 5
beads
P-Tyr
IRS2

Figure S7

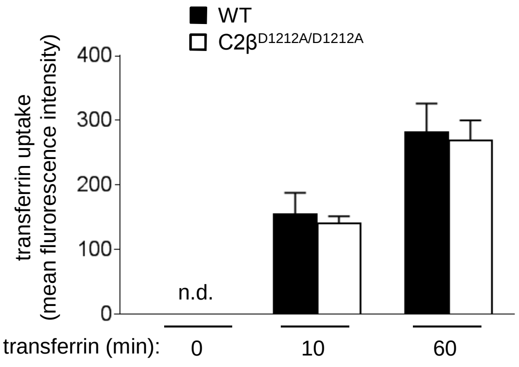
WT
C2β D1212A/D1212A
transferrin uptake
(mean flurorescence intensity)
400
300
200
100
0
n.d.
transferrin (min):
0
10
60

SUPPLEMENTAL DATA

Figure S1. Body Weight of Mice over Time, Related to figure 1.

Figure S2. EGF- or IGF-1-stimulated pAkt-S473 in Hepatocytes, Related to figure 3.
(A) Cultured hepatocytes were starved overnight and stimulated for the indicated time points with EGF, followed by SDS-PAGE analysis and immunoblotting using the indicated antibodies.
(B) Cells were starved and stimulated with IGF-1 as described under (A).
Quantification of data from 3 hepatocyte cell cultures/genotype is shown. Data represent mean ± SEM. *p < 0.05, **p ≤ 0.01, ***p ≤ 0.001.

Figure S3. Impact of PI3K-C2β Inactivation on the Early Stage of Starvation-induced Autophagy in Hepatocytes, Related to figure 5.
(A) Quantification of the size of vesicles stained using GST-2xFYVE^{HRS} or antibodies to APPL1. Representative data from 3-4 hepatocyte cell cultures/genotype are shown.
(B) Analysis of LC3, WIPI-1 and WIPI-2 staining. Hepatocytes were incubated in complete medium or starved for 30 min with EBSS. The inset shows a higher magnification (300x). DAPI-stained nuclei are shown in blue. Quantification was performed using Metamorph software on 3-4 independent hepatocyte cell cultures/genotype. Data represent mean ± SEM. *p < 0.05, **p ≤ 0.01, ***p ≤ 0.001. a.u., arbitrary units. Scale bar, 20 μm.

Figure S4. Impact of PI3K-C2β Inactivation on Early and Late Endosomes in Hepatocytes, Related to figure 5.
Analysis of EEA1 and Rab7 staining in overnight starved hepatocytes. The inset shows a higher magnification (300x) of EEA1-positive vesicles. DAPI-stained nuclei are shown in blue. Quantification was performed using Metamorph software on 3 independent hepatocyte cell cultures/genotype a.u., arbitrary units. Scale bar, 20 μm.
Data represent mean ± SEM. *p < 0.05, **p ≤ 0.01, ***p ≤ 0.001.

Figure S5. Impact of PI3K-C2β Inactivation on IR mRNA Levels in Liver, Related to figure 6.
a.u., arbitrary units.

Figure S6. Impact of PI3K-C2β Inactivation on the tyrosine phosphorylation of IR and IRS proteins, Related to figure 6.
Cultured hepatocytes were serum-starved overnight and stimulated for the indicated times with 100 nM insulin, followed by immunoprecipitation using the indicated antibodies. The immune complexes were analyzed by Western blotting and probed with the indicated antibodies. The results of 4-5 independent experiments (labeled a to e) are shown.

Figure S7. Impact of PI3K-C2β Inactivation on Transferrin Uptake in Hepatocytes, Related to figure 6.
Hepatocytes were starved overnight and stimulated with Alexa Fluor 647-transferrin for the indicated time points. Quantification of data from 3 hepatocyte cell cultures/genotype is shown. n.d., not detected. Data represent mean ± SEM. *p < 0.05, **p ≤ 0.01, ***p ≤ 0.001.

Table S1. List of organs and tissues subjected to histological examination, Related to figure 1.

adrenal	skeletal muscle
aorta	esophagus
brain	optical nerve
brown fat	pancreas
colon	perigenital fat pad
duodenum	perirenal fat pad
epidydimes	pituitary
eyes	prostate
femur	salivary gland
gall bladder	sciatic nerve
harderian gland	skin
heart	spinal cord
jejunum, ileum	spleen
kidney	sternum
liver	stomach
lungs	testis/ovaries
mammary glands	thymus
mesenteric lymph	thyroid
urinary bladder	vagina/uterus

Table S2.Metabolic parameters in WT and C2β$^{D1212A/D1212A}$ mice, Related to figure 2.

**Parameter**	**WT**	**C2β$^{D1212A/D1212A}$**
Leptin (ng/ml)	3.3±0.6	3±0.5
Adiponectin (μg/ml)	10.4±0.5	11.6±1.1
Triglycerides (mg/dl)	58.1±5.7	51±4.3
Free fatty acids (μM)	426.3±26.1	454.4±19.9
Cholesterol (mM)	2.3±0.2	2.1±0.1
Food intake (g/day)	4.4±0.5	3.6±0.5
Energy expenditure (kCal/kg/h)	15.9±2.4	16.5±2.6

SUPPLEMENTAL EXPERIMENTAL PROCEDURES

Metabolic Analysis

For glucose tolerance tests, mice were fasted overnight (16 h) followed by an intraperitoneal injection of glucose (2 g/kg body weight). Blood glucose levels were monitored before and 15, 30, 60, and 100 min after injection using blood collected from tail veins using a Glucotrend glucometer (Roche Diagnostics). For insulin tolerance tests, mice were fasted overnight (16 h), followed by injection with human insulin (0.75 U/kg body weight). Tail blood was collected before and 15, 30, 60 min after injection and glucose levels were determined as described above. For *in vivo* insulin stimulation, mice were fasted overnight (16 h) followed by intraperitoneal injection of insulin (0.75 U/kg body weight) or vehicle (PBS). After 30 min, mice were sacrificed and tissues snap-frozen in liquid nitrogen. Triglyceride levels in liver tissue were determined as described (Peterson et al., 2011). Serum levels of insulin, leptin, triglyceride, cholesterol and adiponectin were measured by ELISA and colorimetric kit (Crystal Chem Inc. for insulin and Millipore for leptin, and adiponectin; Cayman Chemical Company for Triglyceride and, cholesterol). Measurement of oxygen consumption and food intake were obtained with a CLAMS (Columbus Instruments) open-circuit indirect calorimetry system, as described previously (Blouet et al., 2008).

Lipid Kinase Assay

Lipid kinase assay on PI3K-C2β immunoprecipitates using PI as a substrate was performed as previously described (Chaussade et al., 2007).

Western Blot Analysis

Tissues or cells were lysed in 20 mM Tris-HCl (pH 8.0), 5% glycerol, 138 mM NaCl, 2.7 mM KCl, 1% NP-40, 20 mM NaF, 5 mM EDTA, 1 mM sodium orthovanadate, 20 µM leupeptin, 18 µM pepstatin, 4 µg/ml aprotinin, 1 mM DTT. To remove cell debris, homogenates were spun at 13,000 rpm for 10 min at 4°C and the supernatant fraction recovered. Protein concentration was determined by colorimetric assay (Bradford assay, Biorad). Protein extracts were resolved by SDS-PAGE, transferred to PVDF membranes and incubated overnight at 4°C with specific antibodies. Antigen-specific binding of antibodies was visualized by ECL.

Immunofluorescence

Hepatocytes were seeded at 2.5×10^5/well on collagen-coated glass coverslips in 6 well plates as described in the section 'Hepatocyte Isolation and Culture' Cells were fixed with 4% paraformaldehyde and permeabilized with 0.2% Triton X-100 for 5 min. Permeabilized cells were blocked in PBS/2% BSA for 1 h and incubated in PBS/2% BSA with the indicated antibodies at 4°C overnight. After 3 washes with PBS, cells were incubated with species-specific Cy3- or FITC-labeled secondary antibodies for 1 h at room temperature. After 3 washes with PBS, coverslips were mounted on glass slides using Vectashield containing DAPI (Vector Laboratories). Staining with GST-$2xFYVE^{HRS}$ was performed as described (Gillooly et al., 2000), using permeabilization with digitonin as described (Hammond et al., 2009) as follows. Cells were washed in PBS with 2%BSA for 5 min, followed by permeabilization with 20 µM digitonin in PBS with 2%BSA for 5 min at room temperature, followed by 3 washes in PBS with 2% BSA. Cells were incubated with the GST-$2xFYVE^{HRS}$ probe at 0.5 µg/ml for 30 min in PBS with 2% BSA. After 3 washes with PBS with 2% BSA, the cells were incubated with anti-GST antibody for 45 min, with further incubation with secondary antibody reagents as described above. Immunostaining for autophagy markers (LC3 and WIPI-1 and -2) were performed as follows: cells were washed twice with PBS before adding cold methanol (-20°C) for 15 min. Cells were then washed twice with PBS and blocked in 3% BSA/PBS for 1 h and incubated in blocking solution overnight at 4°C with the indicated antibodies. After 3 washes with PBS, cells were incubated with species-specific Cy3- or FITC-labeled secondary antibodies for 1 h at room temperature. After 3 washes with PBS, the coverslips were mounted on glass slides as

described above. All coverslips were analysed using a 63X objective on a 710 Zeiss confocal microscope.
Quantitative analysis of microscopy images was performed using the 'count nuclei' settings application module in the Metamorph software, using a standard algorithm provided by the software. This module allows the identification of individual vesicles by image segmentation and labelling of individual vesicles in different colors. A threshold of a minimum and a maximum width is set to separate vesicles that are close to each other or touching. Signal to noise ratio is defined by adjusting intensity above local background. Any vesicle-like pattern with approximate width below or above this threshold is considered as noise and will be excluded from the analysis.

Immunoprecipitation
Hepatocytes were seeded at $3x10^6$/well on collagen-coated 10 cm plates as described above. After stimulation, cells were lysed in 20 mM Tris-HCl (pH 8.0), 5% glycerol, 138 mM NaCl, 2.7 mM KCl, 1% NP-40, 20 mM NaF, 5 mM EDTA, 1 mM sodium orthovanadate, 20 µM leupeptin, 18 µM pepstatin, 4 µg/ml aprotinin, 1 mM DTT. To remove cell debris, homogenates were spun at 13,000 rpm for 10 min at 4°C and the supernatant fraction recovered. Protein concentration was determined by the Bradford assay. 1 mg of protein was used for immunoprecipitation using the indicated antibodies. The immune complexes were analyzed by western blot and probed with the indicated antibodies.

Determination of PI3P and PIP_3 in Tissue and Cell Extracts
PI3P levels were quantified by a mass assay as previously described (Chicanne et al., 2012). Quantification of PIP_3 by mass spectrometry was performed as previously described (Clark et al., 2011). Preparation of tissues and cell extract for mass assay and mass spectrometry was as follows. After removing media, cells were immediately scraped on ice with ice-cold 1 M HCl, followed by centrifugation at 2000 rpm at 4°C and snap-freezing of the cell pellet. Samples were stored at -80°C before processing for PI3P mass assay or PIP_3 mass spectrometry. For preparation of splenocytes, spleens were isolated from overnight starved mice and put on ice in PBS. The spleens were squeezed through a 70 µm filter to release the cells, followed by centrifugation at 2000 rpm at 4°C and resuspension in red blood cell lysis buffer for 5 min. Cells were washed twice with PBS followed by snap-freezing of the cell pellet. Samples were stored at -80°C before processing for PI3P mass assay.

Insulin Binding and Uptake Assays
Insulin binding capacity was determined as follows. Hepatocytes were seeded at $5x10^5$ cells/well on 6-well plates. Following overnight incubation in starvation medium, cells were incubated on ice for 20 min with 100 nM insulin-FITC, followed by a single wash in ice-cold PBS with 0.2% BSA, 1 mM NaCl, 1 mM $MgCl_2$, followed by 3 washes in PBS with 0.5% BSA. Cells were isolated by gentle scraping off the dish on ice, transferred to FACS tubes and analysed using a BDLSR Fortessa cell analyser. For insulin or transferrin internalisation studies, hepatocytes were seeded as detailed for insulin binding studies. Following overnight incubation in starvation medium, cells were incubated at 37°C with 100 nM FITC-insulin or 20 µg/ml Alexa Fluor 647-transferrin for different time points, after which cells were put on ice to stop ligand internalisation. Cells were then washed once in ice-cold PBS with 0.2% BSA, 1 mM NaCl, 1 mM $MgCl_2$, incubated in acid wash solution (0.2 M NaCl and 0.2 M acetic acid in water) for 5 min, followed by 3 washes in PBS with 0.5% BSA. Cells were isolated by gentle scraping off the dish on ice, transferred to FACS tubes and analysed using a BDLSR Fortessa cell analyser.

Subcellular Fractionation
Primary hepatocytes were starved overnight and stimulated with 100 nM insulin at the indicated time points. Cells were gently scraped off the culture dish and resuspended in ice-cold fractionation buffer [250 mM sucrose, 5 mM EGTA, 20 mM HEPES pH 7.4, phosphatase and protease inhibitor cocktail (Calbiochem)], followed by homogenization by passing 20x through a 25g needle. The

homogenate was centrifuged at 760*g* for 5 min to remove nuclei and unbroken cells. The supernatant was centrifuged at 41,000*g* for 90 min to pellet the crude plasma membrane (CPM). The supernatant contains the light microsome (LM) fraction. Both CPM and LM fractions were resuspended in 1x sample buffer.

Histology

For tissue sections, hematoxylin and eosin (H&E) staining was performed on 5 μm paraffin sections of tissues fixed for 24 h in 4% phosphate-buffered paraformaldehyde (PFA) at 4°C. For Oil Red O staining, liver tissue was frozen in OCT, sectioned, and stained using Oil Red O (Sigma-Aldrich) according to standard procedures. In brief, sections were fixed with 4% PFA in PBS at room temperature for 15 min. Fixed sections were washed again with PBS and stained with Oil Red O (0.5% w/v isopropanol, diluted 3:2 in PBS) for 1 h at room temperature. Stained sections were rinsed in 60% isopropanol, followed by deionized water and mounted in Vectashield (Vector Laboratories).

RNA Extraction and RT-qPCR

Total RNA was prepared from ~30 mg of liver tissue using an RNeasy Mini Kit (Qiagen) according to the manufacturer's instructions. RNA quantity and quality was determined using Nanodrop2000. Single-stranded cDNA was synthesized from 1 µg of total RNA with random hexamer primers and SuperScript II reverse transcriptase (Invitrogen). Real-time quantitative PCR (RT-qPCR) was performed with a Stratagene instrument (Stratagene) according to the manufacturer's instructions using SYBR green PCR Master Mix (Stratagene). We determined the relative amounts of the mRNAs studied by means of the $2^{-\Delta\Delta CT}$ method, with the actin and GAPDH genes as the reference gene and WT samples as the invariant controls for all studies. The results of RT-qPCR are given in arbitrary units.
